# Wearable monitoring during music-based interventions in dementia: physiological and behavioral observations from a pilot study

**DOI:** 10.3389/fnhum.2026.1854021

**Published:** 2026-07-15

**Authors:** Haoran Zhou, Nan Jiang, Santiago Bernheim, Peter Paik, Qiqi Zhou, Katsuo Kurabayashi, Kendra Ray

**Affiliations:** 1 Department of Mechanical and Aerospace Engineering, Tandon School of Engineering, New York University, Brooklyn, NY, United States; 2 Department of Electrical and Computer Engineering, College of Engineering, Carnegie Mellon University, Pittsburgh, PA, United States; 3 Department of Electrical and Computer Engineering, Tandon School of Engineering, New York University, Brooklyn, NY, United States; 4 Department of Rehabilitation Medicine, NYU Langone Health, New York, NY, United States

**Keywords:** Alzheimer's disease and related dementias, music-based intervention, wearable sensing, physiological monitoring, behavioral observation, multimodal dataset, dementia care

## Abstract

**Introduction:**

Music-based interventions (MBIs) are widely used in dementia care, but objective methods for characterizing participant responses during intervention sessions remain limited. Synchronized datasets combining wearable physiological signals and behavioral observations are particularly scarce.

**Methods:**

We conducted a pilot feasibility study involving five individuals with Alzheimer's disease and related dementias (ADRD) who participated in 13 formal MBI sessions. Physiological signals, including photoplethysmography (PPG), electrodermal activity (EDA), skin temperature (TEMP), and accelerometry (ACC), were collected using a wrist-worn wearable sensor and synchronized with intervention playlists and time-stamped behavioral observations. Exploratory analyses examined physiological responses across intervention phases, participant-specific response patterns, time-of-day effects, and music-preference effects.

**Results:**

The dataset contains 13 intervention sessions, 99 music segments, and 248 behavioral observations. PPG, ACC, TEMP, and behavioral observations were available for all sessions, while EDA quality varied because of sensor-contact challenges. Behavioral responses were highly heterogeneous across participants, with engagement and calm behaviors observed most frequently. Physiological responses also showed substantial inter-individual variability, and case studies demonstrated that physiological and behavioral responses were not always concordant.

**Conclusion:**

This study demonstrates the feasibility of collecting synchronized physiological, behavioral, and intervention-context data during MBIs in people living with dementia. The resulting publicly available multimodal dataset provides a foundation for future investigations of participant-specific responses and adaptive music-based interventions.

## 1 Introduction

Music-based interventions (MBIs) are increasingly used in dementia care to support emotional wellbeing, social engagement, and behavioral symptom management in people living with Alzheimer's disease and related dementias (ADRD) (Matziorinis and Koelsch, 2022; Leggieri et al., 2019; King et al., 2019; Peck et al., 2016; Ray and Mittelman, 2017; Bleibel et al., 2023). Despite their widespread adoption, understanding how individuals respond during intervention sessions remains challenging. Therapists and caregivers often rely on behavioral observations and intermittent clinical assessments, while objective and time-synchronized measures of participant responses remain limited.

Current evaluation of MBI relies primarily on clinical questionnaires such as the Cohen-Mansfield Agitation Inventory (CMAI), Clinical Frailty Scale (CFS), and Quality of Life Scale for Alzheimer's Disease (QoL-AD) (McCreedy et al., 2022; Conard et al., 2025; McDermott et al., 2014). Although these measures provide valuable clinical information, they are typically collected intermittently and may be influenced by observer interpretation and reporting bias (Schulz et al., 2013; Pozzi et al., 2023). Furthermore, many people living with dementia (PwD) experience difficulty communicating internal emotional or physiological states (Lee et al., 2013; Warren, 2022), making it challenging for therapists and caregivers to determine how participants respond during intervention sessions using behavioral observations alone. Consequently, they provide limited insight into how PwD respond throughout the course of an intervention session.

Previous studies have also investigated music-related responses using both wearable physiological signals, such as heart rate variability (HRV) and electrodermal activity (EDA), and neurophysiological modalities including electroencephalography, functional magnetic resonance imaging, and positron emission tomography (Van der Steen et al., 2025; Cheng et al., 2022; Nicolini et al., 2024; Loggia et al., 2011; Nagai et al., 2004; Ara and Marco-Pallarés, 2021; King et al., 2019; Jacobsen et al., 2015). While these approaches provide valuable information regarding physiological and neural activity, many require specialized equipment, extensive setup procedures, or offline analysis, limiting their practicality in nursing facilities and other real-world dementia care environments (Hanzal et al., 2023; Kadem et al., 2026).

Recent advances in wearable sensing technologies provide a practical alternative for continuous and unobtrusive monitoring in real-world care settings (Wu et al., 2025; Iaboni et al., 2022; Saif et al., 2020). Wearable sensors can continuously acquire physiological signals while minimizing disruption to intervention activities and participant routines. However, despite growing interest in wearable monitoring, publicly available MBI datasets that integrate physiological recordings, intervention context, and time-synchronized behavioral observations remain scarce. Existing studies often focus on isolated physiological measures or aggregate behavioral outcomes or self-reported scale (Palermo et al., 2023; Ihara et al., 2023), limiting opportunities to investigate how physiological responses and observable behaviors evolve together during MBI. As a result, the feasibility of collecting synchronized multimodal physiological and behavioral data in real-world ADRD care settings remains insufficiently explored.

To address these gaps, we conducted a small-scale pilot feasibility study involving wearable physiological monitoring during MBI sessions in PwD. Physiological recordings were collected together with intervention playlists and time-synchronized behavioral observation notes during real-world intervention sessions. The resulting multimodal dataset enables investigation of relationships among physiological responses, intervention context, and observed participant behaviors across pre-, during-, and post-intervention phases.

The contributions of this work are as follows:

First, we demonstrate the feasibility of collecting synchronized physiological and behavioral data during MBI sessions in PwD using wearable sensors, a custom software backend, and a self-developed iOS application in real-world care settings.

Second, we release a pilot multimodal dataset of five participants consisting of synchronized photoplethysmogram (PPG), EDA, skin temperature (TEMP), and accelerometry signals (ACC), intervention playlists, and time-synchronized behavioral observation notes. Behavioral observations were recorded with fine-grained time stamps ranging from several seconds to approximately one minute and cross-checked by three independent reviewers, enabling alignment of physiological signals, intervention events, and observed participant responses.

Third, we provide exploratory analyses of physiological and behavioral responses across pre-, during-, and post-intervention phases and examine participant-specific response patterns through integrated multimodal assessment. Together, these contributions provide a foundation for future studies investigating multimodal assessment and physiological monitoring during MBI in dementia care.

## 2 Method

### 2.1 Wearable monitoring framework

**Figure 1:**
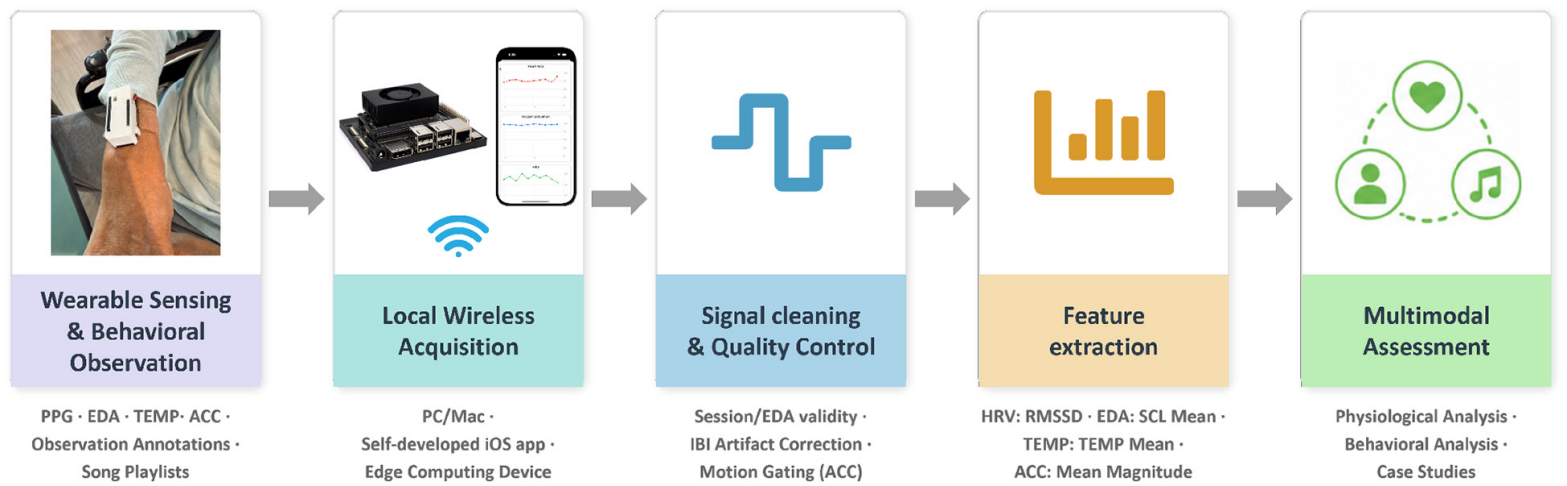
Overview of the wearable monitoring system and data-processing workflow used during MBI sessions. Physiological signals, behavioral observations, and playlist information were synchronized through wearable sensors and local acquisition devices, followed by signal-quality assessment, feature extraction, and multimodal analysis.

The overall wearable sensing and data acquisition and anlysis process is illustrated in Figure 1. A wrist-worn wearable sensor continuously recorded physiological signals and transmitted data through a secure local network to a computing device for visualization, storage, and subsequent analysis. Access to the system was restricted to caregivers and researchers, and all data remained within the facility without external Internet connectivity.

A Python-based backend framework together with a self-developed iOS application was used to receive incoming data streams, synchronize multimodal recordings, visualize sensor signals, and store time-stamped data locally. The iOS application enabled caregivers and researchers to monitor incoming physiological signals, annotate intervention and observation events, and review session status in real time during MBI sessions.

The resulting synchronized data streams formed the basis for both real-time session monitoring and *post-hoc* analyses of physiological and behavioral responses during MBIs.

### 2.2 Subjects

Six individuals with ADRD were recruited from a long-term care facility in Brooklyn, New York, USA. Participant characteristics are summarized in Table 1. None of the participants had previously received systematic MBI using the study protocol. The study was approved by the Salus Institutional Review Board (IRB No. PA-18-591; Study No. 22218) on July 30, 2025, and informed consent was waived in accordance with IRB-approved procedures. Five participants completed at least one formal intervention session and were included in the present analyses. Participant P1 withdrew after the preliminary visit and did not participate in the formal sessions.

**Table 1:** Participant characteristics and study participation status.

Participant	Age	Sex	GDS	Ethnicity	Diagnosis	Status
P0	82	M	6	Black	AD	Four sessions
P1	99	F	6	Caucasian	AD	Withdrawn
P2	80	F	5	White	MCI	Four sessions
P3	77	M	6	White	DLB/Parkinsonism	Two sessions
P4	82	M	6	White	Dementia/MDD	One session
P5	90	M	6	Black	Unspecified dementia	Two sessions

AD, Alzheimer's disease; MCI, mild cognitive impairment; DLB, dementia with Lewy bodies; MDD, major depressive disorder; GDS, Global Deterioration Scale.

### 2.3 Sensor placement and music selection

A preliminary visit was conducted approximately one week before the formal intervention sessions. During this visit, sensor placement procedures were established and participant-specific measurements, including wrist circumference and forearm length, were recorded to promote consistent sensor positioning across sessions.

The wearable sensor was placed on the dominant forearm approximately 10% of the forearm length proximal to the wrist. If the dominant arm could not be comfortably used, the sensor was placed on the contralateral arm. To improve comfort and maintain stable skin contact, particularly for EDA recording, the sensor was secured using a stretchable self-adherent wrap. This approach reduced sensor displacement during routine activities and minimized direct adhesive contact with the skin.

The preliminary visit was also used to determine comfortable listening volumes for MBI sessions. Music selections were determined by a licensed music therapist based on each participant's personal background, music preferences, and prior engagement history. These therapist-informed selections were subsequently incorporated into the intervention playlists used during the formal sessions.

### 2.4 Behavioral observation and annotation

Behavioral observations were collected throughout all formal MBI sessions by an observer team consisting of an experienced nurse/therapist familiar with the participants, an engineer, and a public-health researcher with experience in dementia care. Observers independently documented participant behaviors during the intervention sessions and subsequently reviewed their notes together to produce a consolidated observation record.

Because observations were recorded and reconciled in real time through structured discussion among the three observers, formal inter-rater reliability metrics such as Cohen's κ were not applicable; disagreements were resolved by consensus during each session.

Observation notes included time-stamped descriptions of participant behaviors occurring before, during, and after the intervention. Depending on the event frequency and observer availability, annotations were recorded with temporal resolutions ranging from approximately one minute to several seconds. Because multiple participants were observed simultaneously, some events may not have been captured.

Following each session, observation records were compiled into participant-specific CSV files and synchronized with playlist timestamps and physiological recordings. Behavioral descriptions were subsequently categorized into standardized behavioral labels to facilitate comparison across participants and alignment with physiological measures.

**Figure 2:**
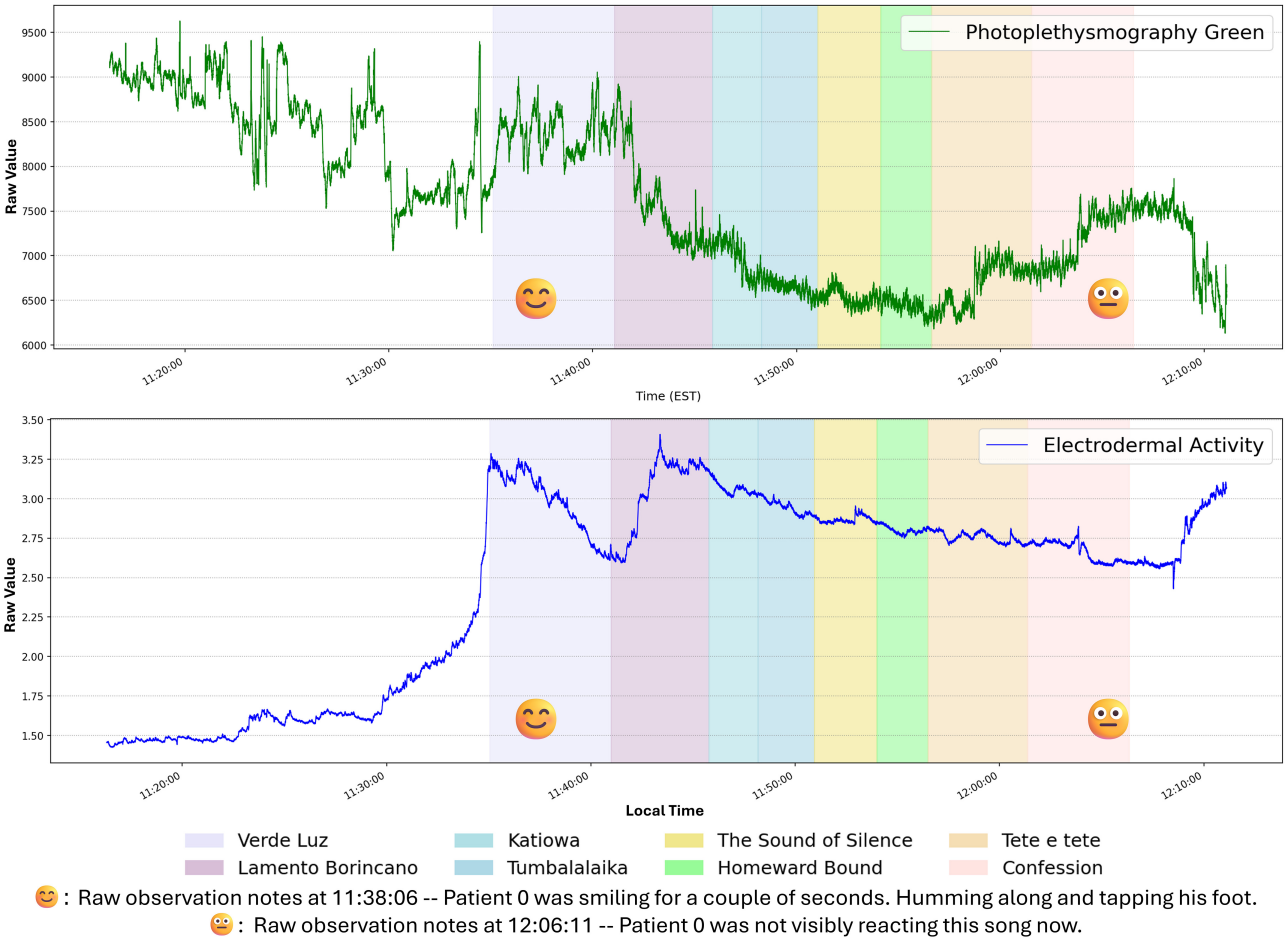
Example of synchronized physiological, intervention-context, and behavioral observation data collected during an MBI session. PPG and EDA recordings are aligned with playlist timestamps and time-stamped behavioral annotations, enabling multimodal analysis of participant responses during music exposure.

An example of synchronized physiological recordings, intervention events, and behavioral annotations is provided in Figure 2.

### 2.5 Data collection during MBI sessions

Each formal MBI session consisted of three phases: *Baseline, Music Intervention*, and *Recovery*.

During the baseline phase, participants engaged in routine activities such as resting, talking, or paper-based activities without listening to music. During the intervention phase, participants listened to a playlist through headphones. All participants attending the same session listened to the same playlist, which was designed by a licensed music therapist familiar with the participants' backgrounds and preferences.

Within each playlist, two songs were designated as preferred songs for each participant based on recommendations from the music therapist and prior engagement history. Because the playlists were shared across participants, a song could be considered preferred for more than one participant. Information regarding playlists and participant-specific preferred songs was documented and included in the released dataset.

Immediately following the intervention phase, participants entered the recovery phase. Headphones were removed, and participants were allowed to rest, converse with caregivers, or interact with other participants.

Session participation is summarized in Table 2. Participants P0 and P2 completed four sessions each, P3 and P5 completed two sessions each, and P4 completed one session. Session durations varied because of differences in participant condition, willingness to participate, and scheduling constraints within the care facility. Baseline duration ranged from approximately 5 min to 2 h. The intervention phase was relatively consistent across sessions and typically consisted of 6–8 songs. Recovery duration was 3 min on Day 1 sessions and 10 min on Day 2 sessions.

**Table 2:** Session completion and signal-quality summary.

Participant	Session day	Session time	Songs	EDA flatness (%)	EDA valid	Motion (%)	Obs. notes
P0	Day 1	Morning	8	9.9	Yes	0.01	13
P0	Day 1	Afternoon	8	2.1	Yes	0.43	12
P0	Day 2	Morning	8	4.1	Yes	0.01	15
P0	Day 2	Afternoon	8	3.7	Yes	0.21	20
P2	Day 1	Morning	8	77.2	No	6.18	22
P2	Day 1	Afternoon	8	1.7	No	0.57	22
P2	Day 2	Morning	7	0.3	No	1.39	28
P2	Day 2	Afternoon	6	63.9	No	1.03	16
P3	Day 2	Morning	6	93.9	No	0.01	22
P3	Day 2	Afternoon	8	94.1	No	0.04	19
P4	Day 1	Morning	8	1.5	Yes	0.00	23
P5	Day 1	Morning	8	91.4	No	0.75	19
P5	Day 1	Afternoon	8	30.8	Yes	0.46	17
Total	13	7 a.m. 6 p.m.	99	36.51 (mean)	6/13	0.853 (mean)	248

EDA validity was determined using baseline EDA quality metrics, including signal flatness and sensor-floor criteria. ACC valid 13/13; PPG valid 13/13; TEMP valid 13/13.

The final dataset contains recordings from five participants across 13 formal intervention sessions and is publicly available through IEEE DataPort (DOI: 10.21227/39y4-s175). The dataset includes de-identified participant identifiers, physiological recordings (PPG, EDA, TEMP, and ACC), playlist information, participant-specific preferred-song labels, and synchronized time-stamped behavioral observation notes.

### 2.6 Data cleaning and processing

Physiological signals collected from the wearable sensor were processed using a custom Python pipeline. Only recordings obtained during the 13 formal MBI sessions were included in the present analyses. Preliminary recordings collected during setup and pilot testing were excluded. Each session was segmented into baseline, music, and recovery phases using synchronized playlist timestamps and manually verified session logs.

#### 2.6.1 PPG processing and RMSSD extraction

PPG signals were sampled at 25 Hz and band-pass filtered using a fourth-order Butterworth filter (0.5–8 Hz) to reduce baseline drift and high-frequency noise (Elgendi et al., 2013). Systolic peaks were detected using the Bishop peak-detection algorithm and used for signal-quality assessment (Bishop and Ercole, 2018; Boloursaz Mashhadi et al., 2016).

HRV analysis was subsequently performed using the sensor-derived inter-beat interval (IBI) channel. Let *IBI*_*i*_ denote the interval between two consecutive heartbeats. Intervals were considered artifactual if the boundary criterion in Equation 1 was met:


$$\begin{array}{c} IBI_{i}<300\text{ ms }\text{or }IBI_{i}>2,000\text{ ms}, \end{array}$$ (1)

or if the relative adjacent-interval difference criterion in Equation 2 was met (Lipponen and Tarvainen, 2019).


$$\begin{array}{c} \frac{\left|IBI_{i}-IBI_{i-1}\right|}{IBI_{i-1}}>0.20 \end{array}$$ (2)

Segments containing fewer than five valid beats, lasting less than 30 s, or containing more than 40% artifactual intervals were excluded.

For valid segments, the root mean square of successive differences (RMSSD) was calculated as shown in Equation 3.


$$\begin{array}{c} RMSSD=\sqrt{\frac{1}{N-1}\overset{N-1}{\underset{i=1}{\sum }}{\left(IBI_{i+1}-IBI_{i}\right)}^{2}}. \end{array}$$ (3)

RMSSD was selected as the primary heart-rate variability metric because it is relatively robust for short-duration recordings and is widely used in wearable-based physiological monitoring studies (Shaffer and Ginsberg, 2017).

#### 2.6.2 EDA processing and skin conductance level

EDA signals were sampled at 15 Hz and processed using NeuroKit2 (Makowski et al., 2021). Signals were low-pass filtered and decomposed into tonic and phasic components (Boucsein, 2012; Makowski et al., 2021).

The mean tonic skin conductance level (SCL) was calculated as shown in Equation 4.


$$\begin{array}{c} SCL=\frac{1}{N}\overset{N}{\underset{i=1}{\sum }}SCL_{i}. \end{array}$$ (4)

Segments shorter than 30 s, containing more than 50% flat samples, or exhibiting median conductance values below 0.035 μS were considered invalid and excluded from EDA analyses, following recommendations from prior EDA signal quality guidelines (Boucsein, 2012).

#### 2.6.3 Accelerometer processing

Tri-axial were sampled at 25 Hz and used both for motion characterization and signal-quality assessment. Acceleration magnitude was calculated as shown in Equation 5.


$$\begin{array}{c} A_{\text{mag}}=\sqrt{A_{x}^{2}+A_{y}^{2}+A_{z}^{2}}. \end{array}$$ (5)

Motion-contaminated samples were identified using the criterion in Equation 6.


$$\begin{array}{c} |A_{\text{mag}}-1g|>0.2g. \end{array}$$ (6)

For each segment, the mean acceleration magnitude was computed as shown in Equation 7.


$$\begin{array}{c} ACC=\frac{1}{N}\overset{N}{\underset{i=1}{\sum }}A_{\text{mag},i}. \end{array}$$ (7)

The resulting ACC feature was used as an exploratory indicator of participant movement during intervention sessions.

#### 2.6.4 Skin temperature processing

Skin temperature signals were sampled at 8 Hz. To reduce transient fluctuations, recordings were smoothed using a moving-average filter. The mean temperature within each analysis segment was calculated as shown in Equation 8.


$$\begin{array}{c} TEMP=\frac{1}{N}\overset{N}{\underset{i=1}{\sum }}T_{i}. \end{array}$$ (8)

Temperature was included as an exploratory physiological measure reflecting peripheral thermal regulation during intervention sessions.

#### 2.6.5 Quality control and missing data handling

Signal quality was assessed independently for each sensing modality. Consequently, a recording could be retained for RMSSD analysis while being excluded from SCL analysis, or vice versa. Excluded segments percentage, missing recordings, and invalid channels were logged and summarized by participant and session. Missing data primarily resulted from insufficient skin contact, sensor detachment, participant withdrawal, early session termination, or recording interruptions.

Several modality-specific quality-control thresholds (e.g., accelerometer-based motion thresholds) were empirically determined during preliminary inspection of the dataset with observation notes and were used to identify recordings affected by poor sensor contact, detachment, or substantial motion artifacts.

#### 2.6.6 Baseline normalization

To facilitate within-participant comparisons, all physiological features were normalized relative to the baseline phase using the logarithmic ratio in Equation 9.


$$\begin{array}{c} x_{\text{norm}}=\ln\;\left(\frac{x_{\text{phase}}}{x_{\text{baseline}}}\right). \end{array}$$ (9)

where *x*_phase_ denotes the feature value during either the music or recovery phase and *x*_baseline_ denotes the corresponding baseline value. Positive values indicate elevations relative to baseline, whereas negative values indicate reductions (Andrianome et al., 2017).

#### 2.6.7 Behavioral observation coding

Behavioral observation notes were collected throughout each intervention session and synchronized with playlist timestamps. Observation records were independently reviewed by three researchers to improve coding consistency.

Because participant behaviors were not mutually exclusive, each observation entry could receive multiple labels simultaneously. Five behavioral categories were defined: *engaged*, indicating active participation (e.g., singing, humming, clapping, drumming, following rhythm, or recognizing songs); *positive*, indicating positive affect (e.g., smiling, laughing, excitement, or positive verbal expressions); *calm*, indicating relaxed or quiet states (e.g., eye closure, stillness, drowsiness, or sleep); *distress*, indicating agitation or discomfort (e.g., anxiety, attempts to leave, shouting, removing the sensor, or ending the session early); and *disengaged*, indicating withdrawal or lack of response (Van Haitsma and Klapper, 1999; Lawton et al., 1996).

A separate quality-control label was used to flag sensor issues, synchronization problems, or recording interruptions; these annotations were not treated as behavioral states but were used to verify data exclusion decisions. Behavioral labels were subsequently aligned with physiological recordings using synchronized timestamps to enable integrated multimodal analysis.

### 2.7 Exploratory statistical analysis

Given the small sample size, heterogeneous participant participation, and repeated-measures structure of the dataset, all analyses were treated as exploratory and hypothesis-generating rather than confirmatory. Formal hypothesis testing was therefore minimized throughout.

For physiological analyses, the session was used as the unit of analysis. Features were averaged within each phase (baseline, music, and recovery) and normalized relative to baseline using the logarithmic ratio described above, yielding one normalized music response and one normalized recovery response per session per feature. Descriptive statistics (means, standard deviations, medians, and interquartile ranges) were computed to summarize responses across sessions.

Behavioral observations were summarized using frequency-based descriptive analyses. Because multiple labels could be assigned to a single observation, behavioral categories were treated as non-mutually exclusive.

Participant-level case studies were conducted by jointly examining physiological trajectories, behavioral observations, and intervention context across individual songs within selected sessions (Ridder et al., 2013). These analyses were intended to illustrate representative response patterns and inter-individual variability rather than support population-level inference.

Additional exploratory analyses examined potential influences of session timing (morning vs. afternoon) and music preference (preferred vs. neutral songs). These analyses were performed for hypothesis generation only and should not be interpreted as evidence of causal or generalized effects.

## 3 Results

### 3.1 Session completion and data quality

Across 13 sessions from five participants, 99 song-level music segments were recorded, corresponding to approximately 325 min of music exposure. Table 2 summarizes session completion and modality-specific data availability by participant and session.

Derived RMSSD, ACC mean, TEMP mean, and behavioral observations were available for all 13 formal sessions. In contrast, EDA availability varied substantially across participants because several recordings showed poor skin contact, excessive flatness, or sensor detachment. EDA was retained for six sessions across three participants: all four sessions from P0, one session from P4, and one session from P5. EDA was excluded for all sessions from P2 and P3 and for one session from P5. These exclusions were consistent with behavioral notes describing sensor removal, poor contact, or early termination.

Overall, the primary missingness occurred in the EDA channel rather than in PPG, ACC, TEMP or behavioral annotations. Motion levels were generally low across formal sessions, with the highest observed motion percentage occurring in P2 Day 1 Morning. These results indicate that synchronized multimodal data collection was feasible, while also highlighting modality-specific signal-quality challenges in real-world PwD care settings.

### 3.2 Observational responses

Behavioral observations were available for all 99 music segments, as summarized in Table 3. Observations were coded using the five-category multi-label scheme: *engaged, positive, calm, distress*, and *disengaged*. Because multiple labels could be assigned to a single observation, category counts were not mutually exclusive.

**Table 3:** Behavioral observation summary across participants.

Participant	Songs	Engaged	Positive	Calm	Distress	Disengaged
P0	32	13	15	6	2	6
P2	29	18	3	16	11	2
P3	14	11	6	6	2	0
P4	8	3	0	5	2	1
P5	16	4	2	9	3	3
All	99	49	26	42	20	12

Labels are multi-label; therefore, counts can exceed the total number of songs.

A total of 248 raw observation notes were recorded across all sessions. Only notes describing participant behavioral or affective states were mapped to the five behavioral categories (Engaged, Positive, Calm, Distress, and Disengaged), resulting in 149 state labels. Remaining notes documented procedural activities, environmental events, subtle movements (e.g., hand motions), or observer coordination/comments and were therefore not included in the behavioral-state counts.

Among the five behavioral categories, *engaged* was the most frequently observed state (*n* = 49), followed by *calm* (*n* = 42) and *positive* affect (*n* = 26). *Distress* and *disengaged* behaviors were observed less frequently, with counts of *n* = 20 and *n* = 12, respectively.

Behavioral response patterns varied substantially across participants. P0 exhibited the highest frequency of positive-affect observations, whereas P2 showed both frequent engagement and the highest number of distress-related observations. P3 demonstrated frequent engagement behaviors with relatively few distress-related observations, while P4 and P5 showed predominantly calm behavioral patterns. These findings highlight substantial inter-individual variability in behavioral responses during MBI sessions and support participant-specific interpretation rather than pooled behavioral conclusions.

### 3.3 Physiological responses

Physiological responses were analyzed at the session level using baseline-normalized log ratios. For each session and physiological feature, the music-phase value was computed as the mean across all available song segments, and the recovery-phase value was computed from the post-music recovery segment. The resulting values were normalized relative to the corresponding baseline phase.

**Figure 3:**
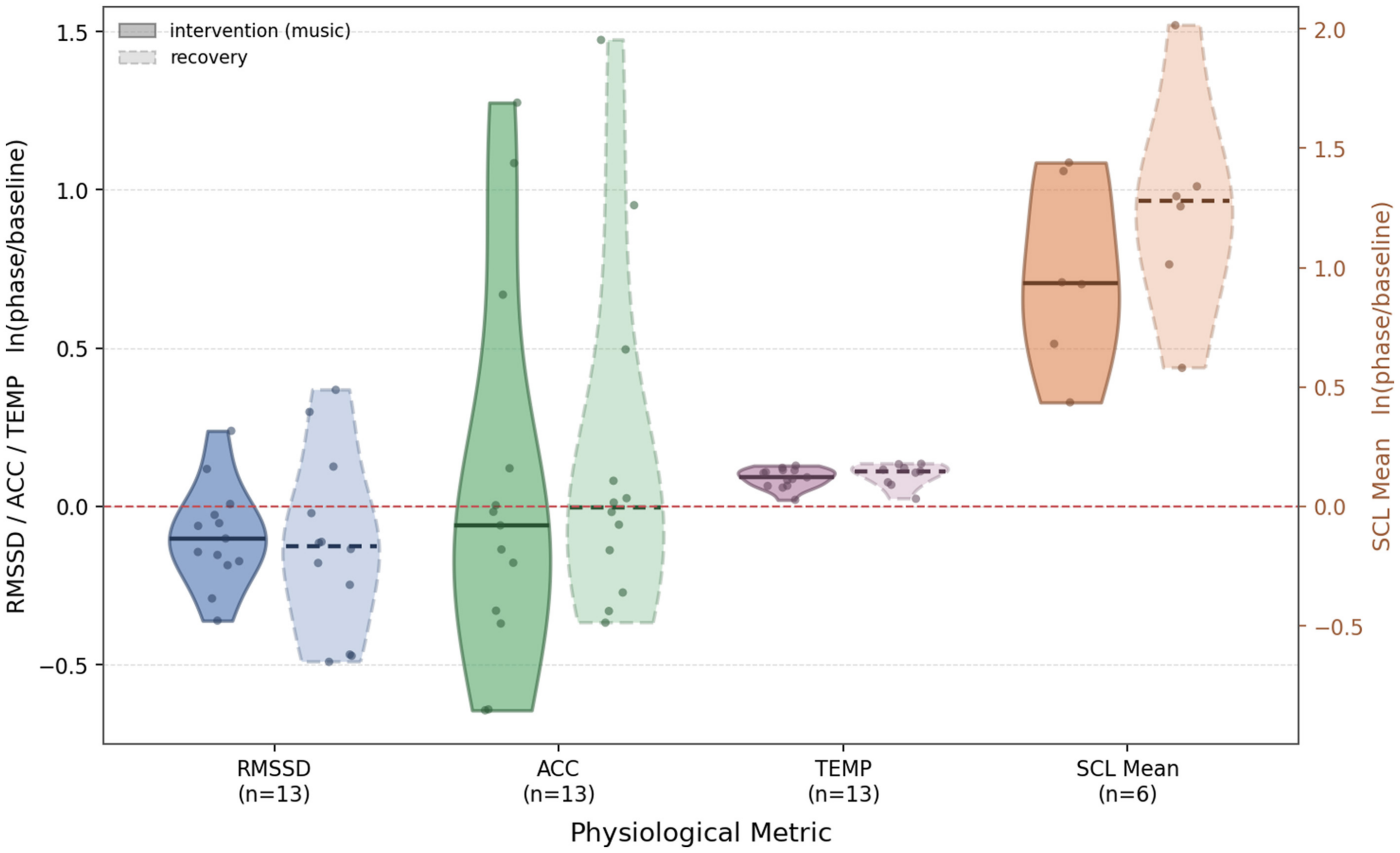
Baseline-normalized physiological responses during intervention (music) and recovery phases. Violin plots show the distributions of RMSSD, ACC, TEMP, and SCL Mean across sessions, with individual session values overlaid. RMSSD, ACC, and TEMP were available for all 13 sessions, whereas SCL Mean was available only for EDA-valid sessions (*n* = 6). The dashed horizontal line indicates the baseline reference (ln = 0).

Figure 3 summarizes the baseline-normalized physiological responses across intervention phases. RMSSD tended to decrease during the music phase and remained below baseline during recovery, indicating a reduction in short-term HRV relative to baseline in many sessions. SCL Mean, available only in EDA-valid sessions, increased during the music phase and remained elevated during recovery. ACC showed substantial variability across sessions, reflecting differences in participant movement and interaction during music exposure. Temperature showed small positive changes across phases, but these values were interpreted descriptively because temperature can be influenced by sensor equilibration and prolonged skin contact.

Importantly, these results were treated as exploratory and descriptive. The observed pattern of RMSSD decrease and SCL Mean increase should not be interpreted as evidence of a specific autonomic state. Instead, it suggests that music exposure was often associated with measurable physiological changes or non-specific arousal [i.e., a general increase in autonomic activation not attributable to a specific emotional state (Critchley, 2002; Berlyne, 1960)], which should be interpreted in conjunction with behavioral observations and participant-level context presented in the following sections.

### 3.4 Combined physiological and observational case-by-case analysis

Two representative sessions are presented in the main text, while additional participant-level examples are provided in the Supplementary material.

**Figure 4:**
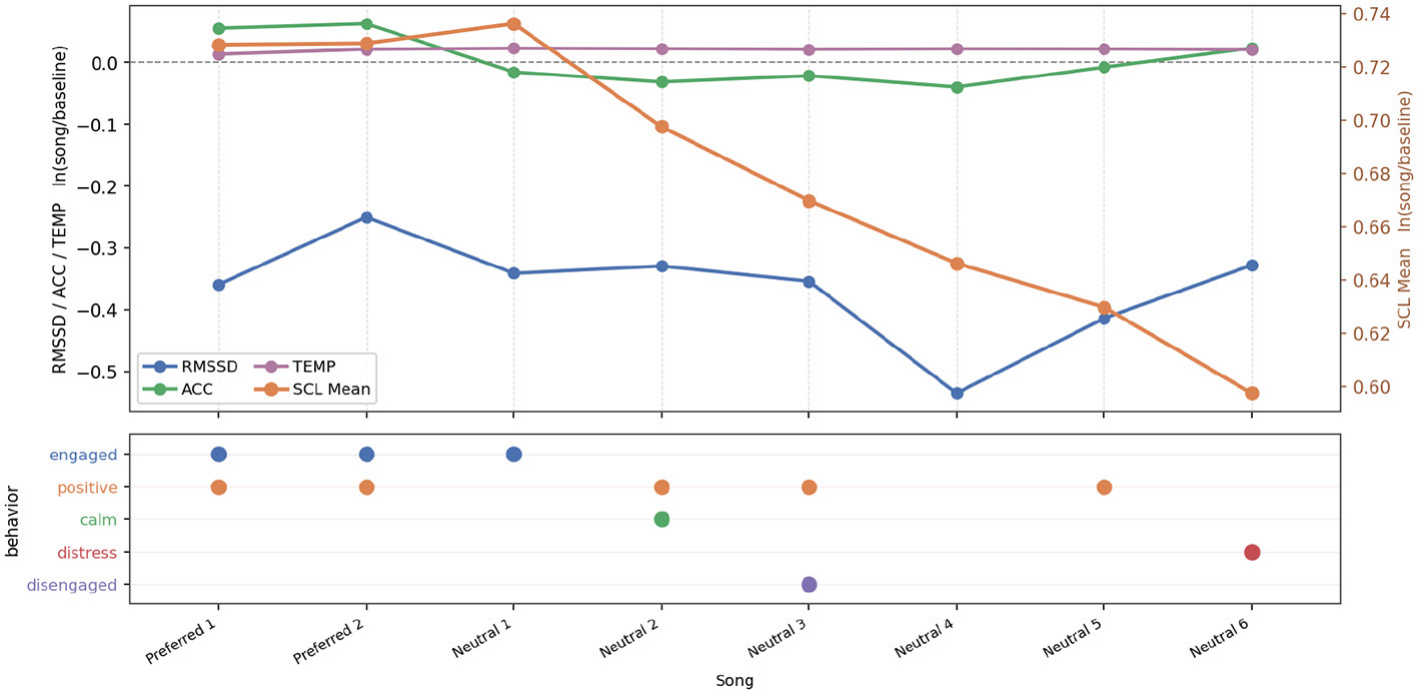
Example participant-level multimodal case study (P0, Day 1 Morning). The upper panel shows baseline-normalized physiological responses (RMSSD, SCL Mean, ACC Mean, and TEMP Mean) across songs, and the lower panel shows synchronized behavioral observation labels. Bold song titles indicate participant-preferred songs.

Participant P0, shown in Figure 4, demonstrated a largely concordant positive response pattern during the Day 1 Morning session. Behavioral observations indicated repeated engagement and positive affect across multiple songs. Physiologically, RMSSD remained below baseline while SCL values were initially elevated relative to baseline but declined progressively across songs. Taken together with the repeated observations of engagement and positive affect, this pattern was interpreted as physiological activation or non-specific arousal occurring alongside positive behavioral engagement. This case illustrates a situation in which physiological and observational measures provided a broadly consistent picture of participant involvement during the intervention.

**Figure 5:**
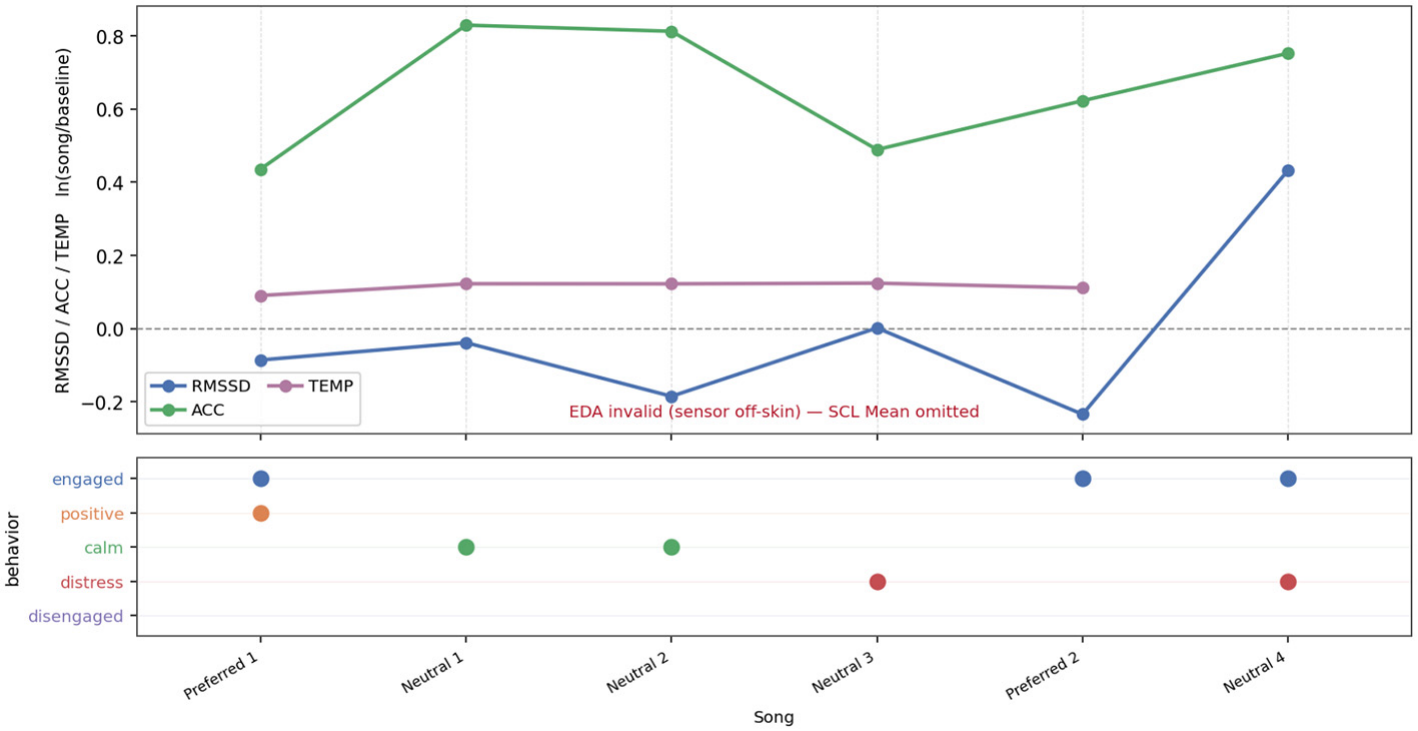
Example participant-level multimodal case study (P2, Day 1 Afternoon). SCL mean is omitted due to EDA unavailability.

Participant P2, shown in Figure 5, exhibited a markedly different response pattern during the Day 2 Afternoon session. Behavioral observations documented visible distress, removal of headphones and sensors, and early termination after six of the eight planned songs. Because EDA recordings were unavailable and RMSSD measurements were likely influenced by movement and recording artifacts (Table 2), interpretation relied primarily on behavioral observations. This case represents a negative response to the intervention and highlights the importance of incorporating contextual and observational information when physiological signal quality is compromised.

Additional participant-level examples are provided in the Supplementary material. Briefly, P3 demonstrated sustained engagement despite unavailable EDA recordings caused by poor sensor contact, illustrating the practical challenges of multimodal physiological monitoring in PwD. P4 exhibited predominantly calm behavioral responses accompanied by elevated and dropped SCL values. This pattern highlights that physiological activation did not necessarily correspond to observable distress and illustrates the importance of interpreting physiological and behavioral measures together. P5 showed mixed behavioral responses, including engagement, calm behavior, disengagement, and repeated distress-related observations later in the session. At the same time, RMSSD remained below baseline throughout most of the intervention. This divergence between physiological and behavioral indicators highlights the complexity of interpreting participant responses using a single modality.

Together, these case studies illustrate the complementary value of multimodal assessment and underscore the need for integrated physiological and behavioral monitoring in real-world MBI research with PwD.

### 3.5 Time-of-day analysis

Time-of-day effects were examined at the session level by comparing morning and afternoon sessions using baseline-normalized music-phase responses. Sessions were pooled across participants for each time-of-day group, as not all participants completed both morning and afternoon sessions. This analysis was therefore exploratory and cannot be interpreted as a within-participant comparison of time-of-day effects (morning: *n* = 7; afternoon: *n* = 6).

**Figure 6:**
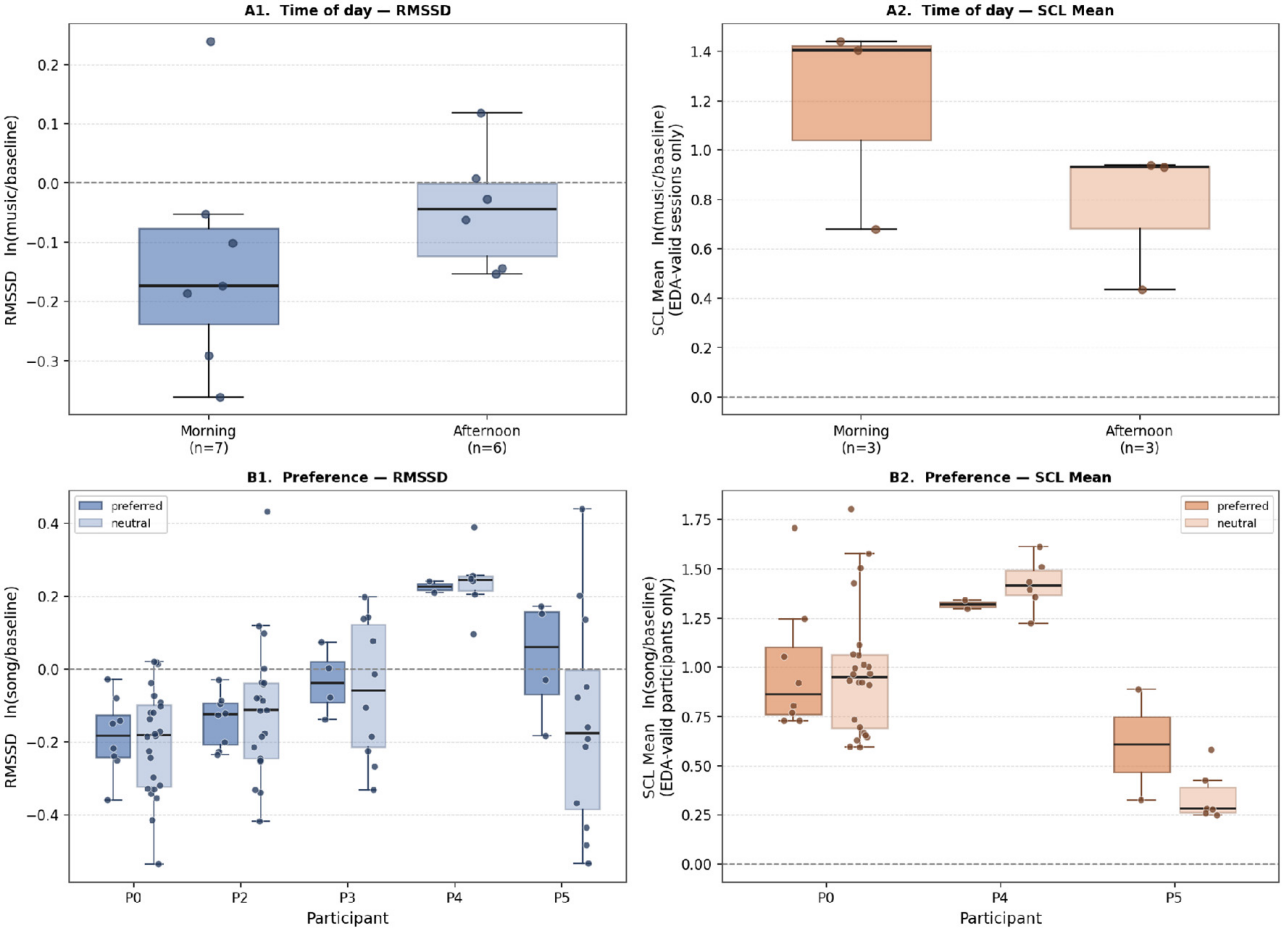
Exploratory analyses of physiological responses during MBI sessions. **(A1, A2)** Session-level distributions of baseline-normalized RMSSD **(A1)** and SCL Mean **(A2)** for morning and afternoon sessions. **(B1, B2)** Song-level distributions of baseline-normalized RMSSD **(B1)** and SCL Mean **(B2)** for participant-preferred and neutral songs. Individual points represent sessions **(A1, A2)** or song segments **(B1, B2)**. SCL Mean analyses were restricted to sessions with valid EDA recordings.

As shown in Figure 6 (A1 and A2), morning sessions generally showed lower RMSSD values relative to baseline (median approximately −0.17), whereas afternoon sessions showed smaller reductions (median approximately −0.04). However, the distributions overlapped substantially, and participant-specific factors cannot be ruled out as contributors to the observed differences.

SCL Mean was evaluated only for EDA-valid sessions (morning: *n* = 3; afternoon: *n* = 3). Morning EDA-valid sessions showed substantially elevated SCL Mean relative to baseline (median ln ratio ≈1.4), while afternoon sessions showed more modest elevations (median ln ratio ≈0.9). Given the small sample size, unequal participant representation across time-of-day groups, and the between-participant pooling, these patterns were interpreted as descriptive observations rather than evidence of a systematic time-of-day effect.

Future studies with larger samples and balanced within-participant designs would be needed to evaluate whether session timing systematically influences physiological responses during MBI.

### 3.6 Music preference

Music preference was analyzed at the song level because preference labels were assigned to individual songs rather than entire sessions. Because songs were nested within sessions and participants, observations cannot be considered statistically independent. Therefore, these analyses were treated as descriptive and exploratory only, and no inferential statistical comparisons were performed. The results are presented to illustrate participant-specific response patterns rather than to support generalized conclusions regarding music preference effects.

As shown in Figure 6 (B1 and B2), RMSSD responses to preferred vs. neutral songs varied considerably across participants. P0 and P2 showed similarly low RMSSD for both preferred and neutral songs, with distributions centered below baseline. P3 showed overlapping distributions near zero, with neutral songs exhibiting wider variability. P4 showed consistently elevated RMSSD for both conditions. P5 showed a higher median RMSSD during preferred songs, though with substantial within-participant variability. No consistent directional preference effect was observed across participants.

SCL Mean was available only for EDA-valid participants (P0, P4, and P5). P0 showed largely overlapping SCL Mean distributions between preferred and neutral songs. P4 showed consistently elevated SCL Mean for both conditions with relatively narrow distributions, indicating little apparent difference between preferred and neutral songs for this participant. P5 showed a higher median SCL Mean during preferred songs compared with neutral songs, though distributions overlapped substantially.

Taken together, these participant-specific patterns suggest that music preference may influence physiological responses in some individuals. However, the small sample size, limited EDA availability, and substantial inter-individual variability preclude generalized conclusions regarding preference-related physiological effects during MBI.

## 4 Discussion

This study evaluated the feasibility of collecting synchronized physiological and observational data during MBI sessions in PwD. The resulting dataset contains wearable recorded PPG, EDA, ACC, TEMP, music context, and time-aligned 248 behavioral observations across 13 sessions and 99 music segments.

The primary finding is that multimodal data collection during routine MBI sessions was feasible in a real-world care environment. Cross checked behavioral observations were available for all sessions, while RMSSD, ACC, and TEMP were successfully acquired across all recordings. In contrast, EDA quality varied substantially, resulting in valid SCL Mean measurements for only a subset of sessions. These findings highlight both the potential and practical challenges of wearable monitoring in PwD during everyday intervention activities.

Behavioral observations revealed considerable heterogeneity across participants. Although engaged and calm were the most frequently observed states overall, participant-specific patterns differed markedly. Some participants showed predominantly engaged and positive responses, whereas others demonstrated calm but withdrawn behaviors or intermittent distress. This variability is consistent with previous MBI literature (Peck et al., 2016; Ray and Mittelman, 2017) suggesting that responses to music are highly individualized in PwD.

Exploratory physiological analyses suggested that RMSSD often decreased during music exposure relative to baseline, whereas SCL Mean tended to increase when valid EDA recordings were available. ACC showed substantial variability across sessions, reflecting differences in participant movement and interaction during music exposure. TEMP exhibited relatively small changes and was therefore interpreted descriptively. Importantly, these physiological responses should not be interpreted as direct indicators of relaxation or therapeutic benefit. Specifically, decreased RMSSD alongside elevated SCL may reflect attentional engagement, orienting responses to auditory stimuli, cognitive activation, novelty responses, or other forms of non-specific autonomic activation during music listening, rather than a specific emotional state such as relaxation or distress (Critchley, 2002; Berlyne, 1960; Cheng et al., 2022; Thayer and Lane, 2000; Jacobsen et al., 2015).

The case studies further demonstrated that physiological and observational responses were not always aligned. For example, some participants exhibited decreased RMSSD despite positive engagement, whereas others displayed elevated SCL Mean during calm behavioral states. These observations suggest that physiological measures alone may not adequately characterize responses to MBI in PwD. Instead, wearable signals should be interpreted together with behavioral observations and intervention context.

A major contribution of this work is the creation of a synchronized multimodal dataset that combines physiological signals, music exposure information, and structured behavioral annotations. Such datasets remain relatively limited in dementia-care research and may support future studies investigating participant-specific responses, multimodal monitoring strategies, and adaptive intervention systems.

Several limitations should be acknowledged. The sample size was small and session participation varied across individuals. The study was observational and exploratory, lacking a control condition, randomization, or counterbalancing procedures; therefore, causal relationships between music exposure, behavioral observations, and physiological responses cannot be established. Behavioral observations relied on qualitative coding by trained researchers and may be subject to inter-rater variability despite cross-checking. Clinical factors including medication use and comorbidities were not systematically controlled and may have influenced physiological responses. Song order was not randomized, and carry-over effects between consecutive songs cannot be excluded. Finally, EDA data quality was inconsistent due to poor skin contact, sensor displacement, and participant removal or manipulation of the device, limiting the sessions available for SCL Mean analysis.

Future work may benefit from alternative sensor placements, improved fixation strategies, and hardware designs that reduce sensor displacement and participant manipulation. Such approaches may improve EDA signal quality and increase the feasibility of long-term physiological monitoring in PwD.

Despite these limitations, this study demonstrates the feasibility of collecting synchronized physiological and observational data during MBI sessions in PwD. The dataset provides a foundation for future investigations of participant-specific responses to music and may support the development and evaluation of multimodal monitoring frameworks for adaptive MBI in dementia care.

## Data Availability

The datasets presented in this study can be found in online repositories. The names of the repository/repositories and accession number(s) can be found below: the dataset supporting the conclusions of this article, titled “A Multi-Modal Physiological and Behavioral Dataset Collected During Music-Based Interventions in Individuals with ADRD,” is publicly available on IEEE DataPort at https://dx.doi.org/10.21227/39y4-s175.
