# Supplementary material for: Wearable monitoring during music-based interventions in dementia: physiological and behavioral observations from a pilot study
Source: Front Hum Neurosci. 2026 Jul 15;20:1854021. doi: 10.3389/fnhum.2026.1854021 (PMC13416265; doi:10.3389/fnhum.2026.1854021)
Supplement: Supplementary file 1 [file Data_Sheet_1.pdf]

Supplementary Material

1 SUPPLEMENTARY FIGURES

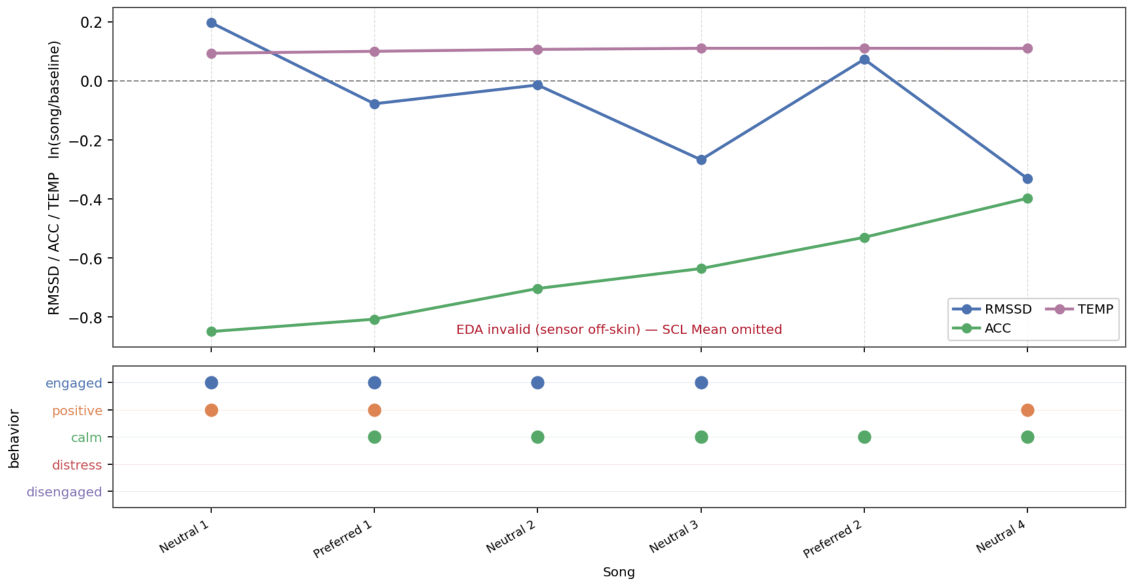

Figure S1. Example participant-level multimodal case study (P3, Day 2 Morning).

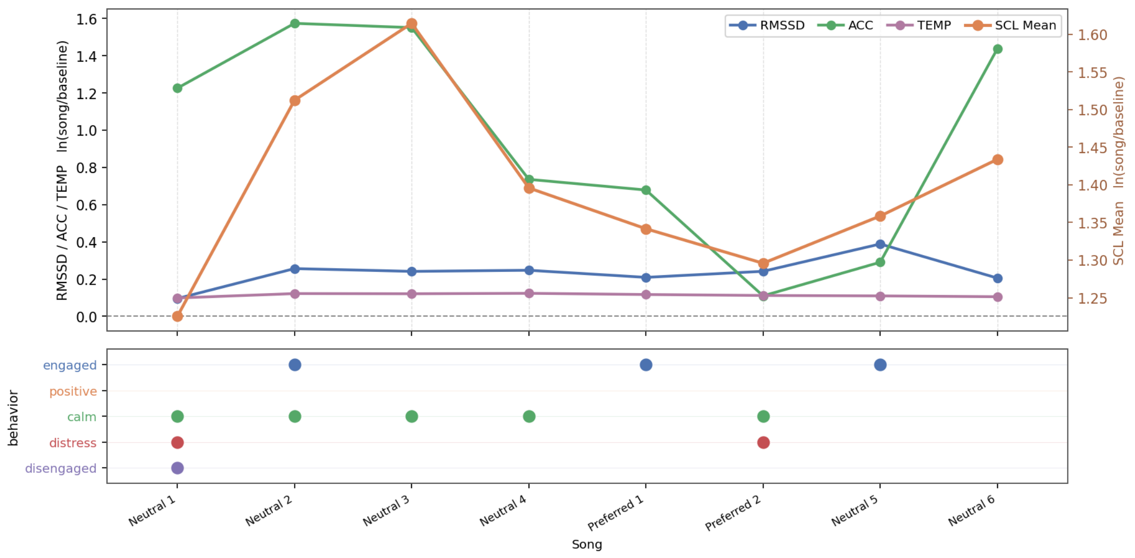

Figure S2. Example participant-level multimodal case study (P4, Day 1 Morning).

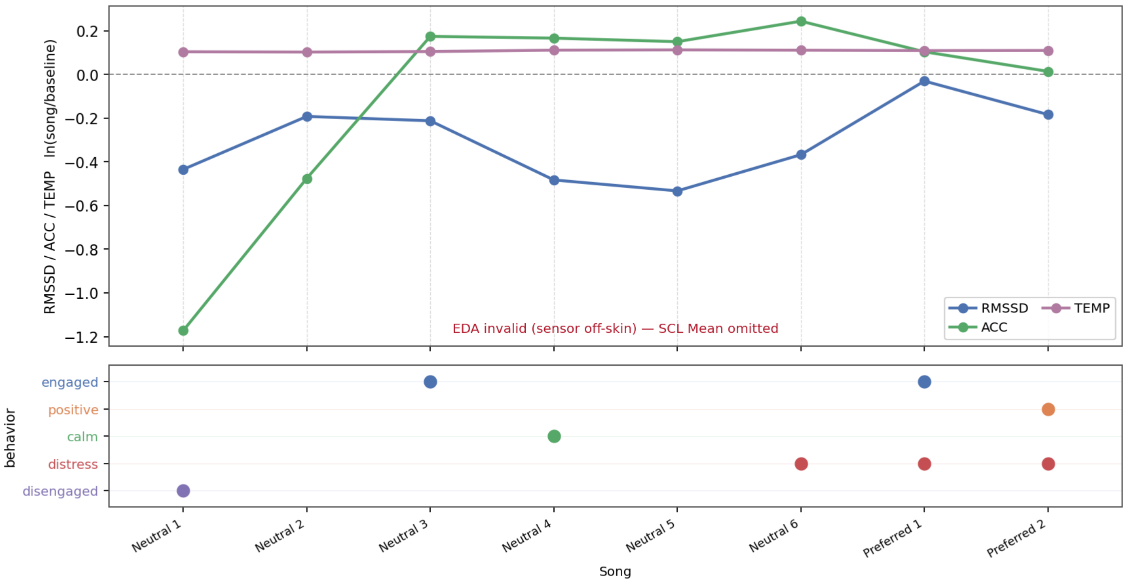

Figure S3. Example participant-level multimodal case study (P5, Day 1 Morning).
